# Stereotactic brain injection of human umbilical cord blood mesenchymal stem cells in patients with Alzheimer's disease dementia: A phase 1 clinical trial

**DOI:** 10.1016/j.trci.2015.06.007

**Published:** 2015-07-26

**Authors:** Hee Jin Kim, Sang Won Seo, Jong Wook Chang, Jung Il Lee, Chi Hun Kim, Juhee Chin, Soo Jin Choi, Hunki Kwon, Hyuk Jin Yun, Jong Min Lee, Sung Tae Kim, Yearn Seong Choe, Kyung-Han Lee, Duk L. Na

**Affiliations:** a Department of Neurology, Samsung Medical Center, Sungkyunkwan University School of Medicine, Seoul, 06351, Korea; b Neuroscience Center, Samsung Medical Center, Seoul, Korea; c Department of Clinical Research Design & Evaluation, SAIHST, Sungkyunkwan University, Seoul, Korea; d Stem Cell & Regenerative Medicine Center, Research Institute for Future Medicine, Samsung Medical Center, Seoul, Korea; e Biomedical Research Institute, MEDIPOST Co., Ltd, Seoul, Korea; f Department of Neurosurgery, Samsung Medical Center, Sungkyunkwan University School of Medicine, Seoul, Korea; g Department of Psychology, Behavioural and Clinical Neuroscience Institute, University of Cambridge, Cambridge, UK; h Department of Biomedical Engineering, Hanyang University, Seoul, Korea; i Department of Radiology, Samsung Medical Center, Sungkyunkwan University School of Medicine, Seoul, Korea; j Department of Nuclear Medicine, Samsung Medical Center, Sungkyunkwan University School of Medicine, Seoul, Korea; k Department of Health Sciences and Technology, SAIHST, Sungkyunkwan University, Seoul, Korea

**Keywords:** Alzheimer's disease, Mesenchymal stem cell, Stereotactic injection, Hippocampus, Precuneus

## Abstract

**Introduction:**

We conducted a phase 1 clinical trial in nine patients with mild-to-moderate Alzheimer's disease to evaluate the safety and dose-limiting toxicity of stereotactic brain injection of human umbilical cord blood–derived mesenchymal stem cells (hUCB-MSCs).

**Methods:**

The low- (n = 3) and high-dose (n = 6) groups received a total of 3.0 × 10^6^ cells/60 μL and 6.0 × 10^6^ cells/60 μL, respectively, into the bilateral hippocampi and right precuneus.

**Results:**

No patient showed serious adverse events including fever during the 24-month follow-up period. During the 12-week follow-up period, the most common acute adverse event was wound pain from the surgical procedure (n = 9), followed by headache (n = 4), dizziness (n = 3), and postoperative delirium (n = 3). There was no dose-limiting toxicity.

**Discussion:**

Administration of hUCB-MSCs into the hippocampus and precuneus by stereotactic injection was feasible, safe, and well tolerated. Further trials are warranted to test the efficacy.

**Clinical Trial Registration:**

ClinicalTrial.gov identifier NCT01297218 and NCT01696591.

## 1 Introduction

Alzheimer's disease (AD) dementia is a neurodegenerative disease that results in progressive dementia. Currently, no approved disease-modifying treatments are available for AD. Mesenchymal stem cells (MSCs) are multipotent stem cells that are capable of self-renewal and differentiation into various cell types when cultured under appropriate conditions [1]. However, it is known that MSCs are less likely to differentiate into neurons when injected into the brain. Rather, MSCs secrete various cytotropic factors that may exert beneficial effects in AD mice through various mechanisms such as reducing amyloid burden, decreasing inflammation,or increasing endogenous neurogenesis [2], [3], [4]. Several human clinical trials have suggested that MSCs are effective in slowing down the course of neurodegenerative diseases such as Parkinson's disease [5], multiple system atrophy [6], and amyotrophic lateral sclerosis [7], [8]. However, to the best of our knowledge, there has been no clinical trial that has attempted to treat human AD using MSCs.

Given the lack of an effective regimen for AD dementia, more innovative treatments are needed to effectively alter the course of the disease. There has been little evidence regarding the ability of MSCs, injected either intraarterially or intravenously, to penetrate through the blood-brain barrier for engraftment into the brain parenchyma of AD dementia patients. Thus, to test the therapeutic potentials of MSCs, we directly transplanted MSCs into the brains of human AD patients using stereotactic surgery because the most effective route of delivering MSCs into a targeted structure may be through direct implantation. Indeed, a recent phase 1 clinical trial reported that the stereotactic injection of nerve grow factor into the nucleus basalis of Meynert of AD dementia patients is both feasible and well tolerated [9].

In the present study, we targeted the hippocampus and precuneus as injection sites because they are areas that are predominantly affected during the earlier phases of AD dementia. According to pathologic and imaging studies, the precuneus is where amyloid start to accumulate in the course of AD [10], [11] and the hippocampus is where neurofibrillary tangles begin to aggregate during the progression of AD [12]. Consistent with these data, recent functional neuroimaging studies have suggested a central role of the precuneus in memory [13] and that decreased hippocampus-precuneus functional connectivity is an early sign of AD [14]. Furthermore, the hippocampus and precuneus undergo atrophy in the early stages of AD dementia [15].

MSCs can be isolated from the umbilical cord blood, which have been widely used in various clinical settings [16], [17], [18]. Establishing the safety and feasibility of a surgical method to effectively deliver human umbilical cord blood–derived MSCs (hUCB-MSCs) into the hippocampus and precuneus would represent an important milestone in advancing the application of this novel treatment for AD dementia. Therefore, the aim of this study was to evaluate the safety and tolerability of surgical stereotactic injection of hUCB-MSCs into the bilateral hippocampus and right precuneus and to assess the maximum tolerated dose. We also investigated the potential efficacy of hUCB-MSCs in AD patients using cognitive measurements and imaging markers.

## 2 Methods

### 2.1 Study design

This was an open-label, single-center, phase 1 clinical trial performed at Samsung Medical Center. The first three AD dementia patients received a low dose (a total of 3.0 × 10^6^ cells, 1.0 × 10^6^ in each side of the hippocampus and 1.0 × 10^6^ in the right precuneus) of hUCB-MSCs. After we confirmed that there were no serious adverse events, additional six AD dementia patients were selected to receive a high dose (a total of 6.0 × 10^6^ cells, 2.0 × 10^6^ in each side of the hippocampus and 2.0 × 10^6^ in the right precuneus) of hUCB-MSCs via the same route. We injected MSCs into only the right precuneus to compare the change in amyloid burden level in the MSC-treated right precuneus with that in the untreated left precuneus. This trial was registered at ClinicalTrial.gov (ClinicalTrial.gov number NCT01297218 for 12 weeks of follow-up; NCT01696591 for extended follow-up of 24 months). We obtained written informed consent from every patient or their legally authorized representatives in cases of impaired capacity. This study was approved by the Institutional Review Board of Samsung Medical vCenter.

### 2.2 Participants

Eligible patients were aged 50–75 years, fulfilled the criteria for probable AD dementia according to the National Institute of Neurological and Communicative Disorders Stroke and AD and Related disorders Association [19], and had a mini-mental state examination (MMSE) score between 10 and 24. Patients with neurologic diseases other than AD dementia were excluded, as were those with one or more of the following conditions: severe white matter hyperintensities on fluid-attenuated inversion recovery (FLAIR) images at baseline, which were defined as a cap or band (periventricular white matter hyperintensities) ≥10 mm, and deep white matter lesions (deep white matter hyperintensities) ≥25 mm as modified from the Fazekas ischemia criteria [20]; major psychiatric disorder; history of stroke within 3 months of enrollment; hepatic, renal, hematologic, or active pulmonary disorder; history of alcohol abuse; and/or underlying malignancy. Patients were required to have been on a stable dose of acetylcholinesterase inhibitors or memantine for at least 60 days before enrollment, and the same dose of medication was continued throughout the study. Nine patients who met the mentioned criteria were also evaluated for amyloid burden using ^11^C-labeled Pittsburgh compound B (PiB) positron emission tomography (PET) as well as the downstream neuronal degeneration biomarker using [^18^F]fluoro-2-deoxy-D-glucose (FDG) PET and structural brain magnetic resonance imaging (MRI). All nine patients were positive for amyloid (standardized uptake value ratio [SUVR] ≥1.5), had decreased FDG uptake in the temporoparietal cortex, and disproportionate atrophy in the medial, basal, lateral temporal lobe, and parietal cortex. Therefore, all the enrolled patients satisfied the research criteria for high likelihood of AD with biomarker evidence for amyloid and neurodegeneration as proposed by the National Institute on Aging-Alzheimer's Association workgroup [21].

The baseline characteristics of each individual subject are listed in Table 1. The mean age was 57.0 years in the low-dose group and 64.0 years in the high-dose group.

**Table 1:** Baseline characteristics of participants

Subject	Age	Gender	Education, y	Medication	MMSE	ADAS-Cog	PiB SUVR
LD01	59	Male	10	AChE-I	23	18	2.7
LD02	58	Male	18	AChE-I and memantine	12	51	2.17
LD03	54	Male	6	AChE-I and memantine	10	47	2.27
HD01	74	Male	16	AChE-I and memantine	18	36	2.53
HD02	62	Male	18	AChE-I	17	30	1.51
HD03	74	Male	12	AChE-I	20	21	2.27
HD04	58	Female	16	AChE-I	12	37	2.51
HD05	60	Female	9	AChE-I and memantine	18	29	2.17
HD06	56	Female	12	AChE-I	20	15	2.28

Abbreviations: MMSE, mini-mental state examination; ADAS-Cog, Alzheimer's disease assessment scale-cognitive subscale; PiB SUVR, Pittsburgh compound B standardized uptake value ratio; LD, low dose; AChE-I, acetylcholine esterase inhibitor; HD, high dose.

### 2.3 Preparation of hUCB-MSCs

To be used for clinical purposes, hUCB-MSCs were manufactured in compliance with Korea Good Manufacturing Practices (KGCP) standards. Cell quality control and quality assurance were performed in compliance with KGCP standards. hUCB tissue was obtained after receiving written informed consent from normal women in their full-term pregnancy. hUCB-MSCs were grown in α-minimum essential medium (α-MEM; Gibco/Life Technologies, Carlsbad, CA, USA) supplemented with 10% fetal bovine serum (Gibco/Life Technologies). Cells were cryopreserved at −150°C or lower using 10% dimethyl sulfoxide.

To prepare for surgical injection, frozen hUCB-MSCs were first thawed, seeded, and cultured. The cells were harvested 5 days after seeding, repeatedly washed to remove impurities such as fetal bovine serum and trypsin, and resuspended in an appropriate amount of phenol red-free α-MEM. Therefore, fetal bovine serum is not present in the final drug product, NEUROSTEM. hUCB-MSCs were tested for viability, phenotype, and presence of endotoxins, bacteria, and mycoplasma. After testing, 50 million cells were resuspended in 1 mL of phenol red-free α-MEM and of the 1 mL, 60 μL was used to inject to each patient in the low-dose group. For the high-dose group, from the adjusted final concentration of 100 million cells per 1 mL of phenol red-free MEM-α, an equivalent volume of 60 μL was prepared for administration into each individual patient in the high-dose group. These samples were then delivered to the patient's physician. hUCB-MSCs were stored at 2–8°C and had a shelf life of about 44 hours from the time of manufacture. Using flow cytometric analyses, the expressions of surface antigens of the cells were consistently positive for CD73, CD90, CD105, and CD166 but negative for CD45, CD14, and HLA-DR.

### 2.4 Stereotactic administration of hUCB-MSCs

Patients underwent perioperative stereotaxic MRI to localize the left and right hippocampi as well as the right precuneus. Intraoperative navigation–guided stereotactic injection was performed by the same surgeon under general anesthesia. Frameless stereotaxis and the BrainLab system were used to inject hUCB-MSCs to the target point. We used a custom-made guiding cannula with a 1.3-mm outer diameter and a 1.1-mm inner diameter. The cannula had a blunt stylet within the lumen to avoid coring when being passed through the parenchyma. The guiding cannula was deigned to expose the distal 10 mm of a Hamilton syringe needle. When the cannula tip reached the target, the stylet was taken out and the absence of bleeding or cerebrospinal fluid (CSF) leak was confirmed. Then the cannula was retracted 10 mm, and then a Hamilton syringe (model 702) with a 22-gauge needle was inserted through the cannula. After reaching the cannula tip, the needle was further advanced 10 mm to reach the target point. We manually injected hUCB-MSCs at approximately 5 μL/5 min at each site. The injection needle was left in place for 5 minutes and then slowly retracted 5 mm for the next site injection.

**Fig. 1:**
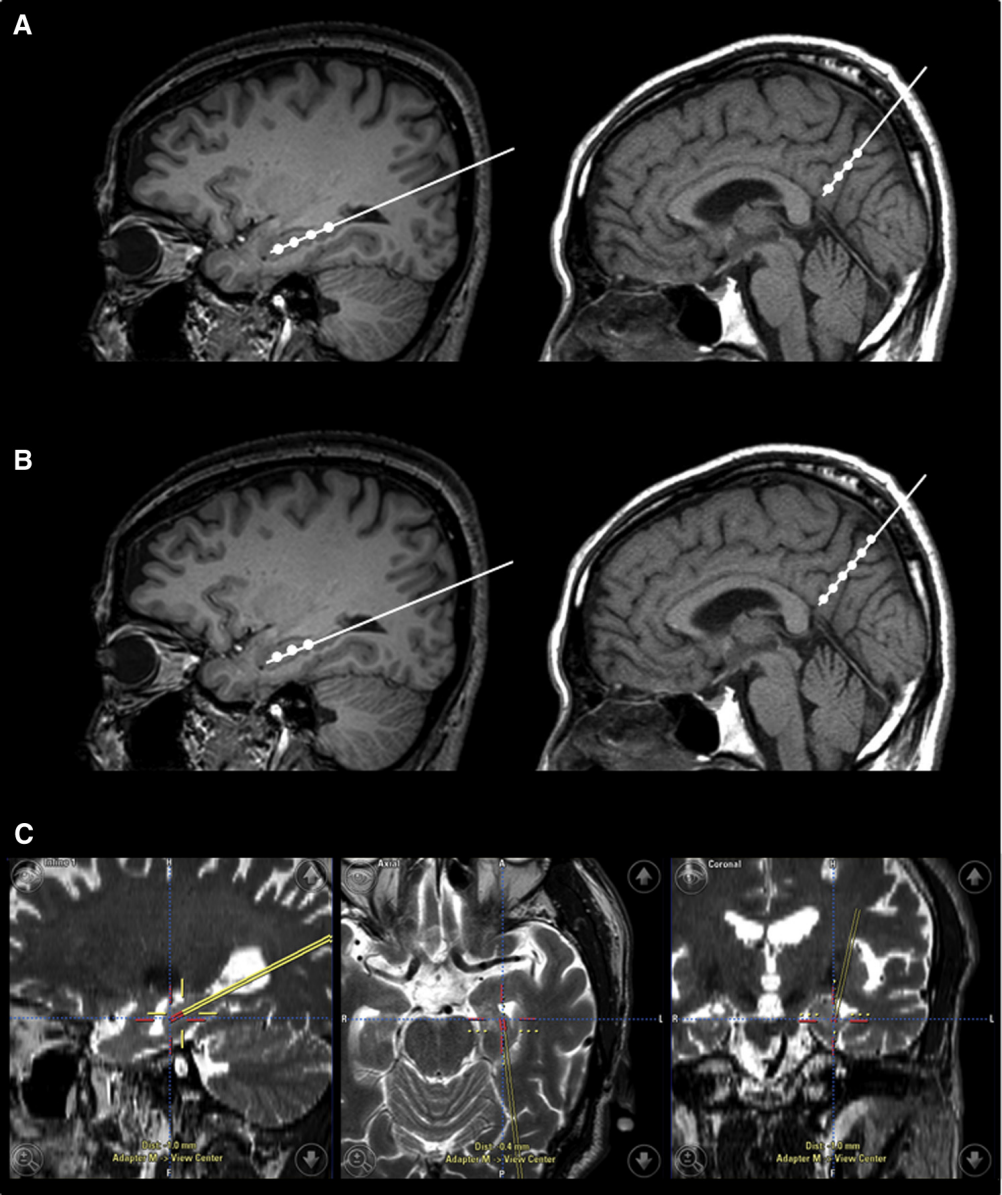
Intraoperative navigation–guided stereotactic administration of hUCB-MSCs. hUCB-MSCs were injected into the right and left hippocampus and right precuneus at four sites (5-mm intervals) along the inserted trajectory while retracting the cannula (A). However, in six of nine patients, the fourth injection was located on the temporal horn of the lateral ventricle, probably due to substantial hippocampal atrophy. In these cases, the omitted doses were injected into the right precuneus, resulting in three injection sites in each hippocampus and six sites in the right precuneus (B). An example of stereotactic administration of hUCB-MSCs into the left hippocampus (C). Each patient received 12 injections for a total of 3.0 × 10^6^ cells/60 μL in the low dose group or 6.0 × 10^6^ cells/60 μL in the high dose group. Abbreviation: hUCB-MSCs, human umbilical cord blood–derived mesenchymal stem cells.

For hippocampal injection, the cannula was inserted through burr holes on each side parallel to the temporal horn border of the lateral ventricle to the mid portion of the hippocampal body, in which the tip was placed 5-mm posterior to the border of lateral ventricle (Fig. 1C). As illustrated in Fig. 1A, each trajectory (left and right hippocampi and right precuneus) consisted of four injection sites. That is, hUCB-MSCs (2.5 × 10^5^ cells/5 μL per site for the low dose and 5.0 × 10^5^ cells/5 μL per site for the high dose) were injected at four sites (5-mm intervals) along the inserted trajectory for both the left and right hippocampi. For precuneus injection, the cannula was inserted perpendicular to the skull at a depth of 5.0 cm. hUCB-MSCs (2.5 × 10^5^ cells/5 μL per site for the low dose and 5.0 × 10^5^ cells/5 μL per site for the high dose) were injected at four sites (5-mm intervals) along the inserted trajectory for the right precuneus (Fig. 1A). Thus, each patient was injected with a total of 3.0 × 10^6^ cells suspended in 60 μL for the low-dose condition or 6.0 × 10^6^ cells in 60 μL for the high-dose condition. For the hippocampal injections, hUCB-MSCs were implanted into the four designated sites as the cannula was retracted. However, in six of nine patients, the fourth injection was located on the temporal horn of the lateral ventricle, which was not possible to inject, likely due to substantial hippocampal atrophy. In these cases, we omitted the fourth dose, only injecting in to a total of three sites for each hippocampus, and instead injected the omitted doses into the right precuneus resulting in a total of six sites as illustrated in Fig. 1B. Although injecting the missed hippocampal dose into the precuneus would lead to a potential bias when comparing the efficacy between low-dose and high-dose groups, this method was necessary to reduce bias in terms of assessing safety and dose-limiting toxicity, which was our primary goal. Participants were hospitalized for 4–6 days after surgical injection and were observed for signs of acute adverse events.

### 2.5 Safety assessments

Our primary goal was to assess the safety and dose-limiting toxicity of two ascending doses of hUCB-MSCs. Patients were assessed at baseline; 1, 2, 4, 7, and 14 days; 4, 8, and 12 weeks; and 12, 18, and 24 months after hUCB-MSC injection. Safety was evaluated by examining vital signs, weight, physical and neurologic examinations, chest x-rays, and clinical laboratory testing (hematologic and serum chemical testing, and urinalysis). Adverse events were assessed at each visit. Acrovan Co, Ltd (Anyang, Korea) served as an external monitor of the study.

To detect immunologic reactions between the implanted hUCB-MSCs and the recipient, a mixed lymphocyte reaction was assessed at baseline and at 12 weeks and 12 months after injection. To detect structural abnormalities including brain tumor or hemorrhage, patients underwent brain computed tomography (CT) 24 hours after surgical injection and brain MRI at 12 weeks and 24 months after injection.

### 2.6 Clinical and laboratory assessments

To measure clinical changes, patients underwent AD assessment scale-cognitive subscale (ADAS-Cog) testing, Seoul instrumental activities of daily living (S-IADL), MMSE, and caregiver-administered neuropsychiatric inventory at baseline and again at 4 weeks, 12 weeks, and 24 months after hUCB-MSC injection.

To measure changes in parenchymal amyloid deposition, patients underwent PiB-PET at baseline and again at 4 and 12 weeks after hUCB-MSC injection, and changes in SUVR were assessed. To measure metabolic changes, patients underwent FDG-PET at baseline and at 12 weeks after hUCB-MSC injection. To evaluate changes in CSF biomarkers, CSF amyloid-β, total-tau, and phosphorylated-tau were evaluated at baseline, 4, and 12 weeks after hUCB-MSC injection. CSF was collected in a sterile polypropylene tube with a conical bottom through lumbar puncture at L3–L5 level using a 22-gauge needle, as recommended by Alzheimer's Biomarkers Standardization Initiative [22]. Details of the imaging procedures for PiB-PET and FDG-PET are provided in the Supplementary Material, available online.

### 2.7 Statistical analysis

Because of the small number of participants, absence of sham surgery control, and the fact that it was an open-labeled study design, we did not perform statistical analysis to compare the outcome measures of the low- and high-dose hUCB-MSC recipients.

## 3 Results

### 3.1 Compliance and safety

All the enrolled patients completed the 12-week follow-up trial; however, during the 24-month extended follow-up study, one patient was excluded due to revocation of consent. hUCB-MSCs injected through the stereotaxic method was completed safely in all nine subjects. Brain CT scans taken within 24 hours after injection confirmed that there was no cerebral hemorrhage in any of the patients. Vital signs and weights were stable, and there was no incidence of fever in any of the patients. Likewise, there were no treatment-related alterations in hematologic parameters. The most common acute adverse event (during the 12-week follow-up) was wound pain from the surgical procedure 9/9 (100%), followed by headache 4/9 (44.4%), dizziness 3/9 (33.3%), delirium 3/9 (33.3%), nausea 2/9 (22.2%), and back pain 2/9 (22.2%; Table 2); however, none of these adverse events were considered serious. There was no adverse event during the 24-month extended follow-up study. Furthermore, all the noticeable adverse events were deemed by investigators as unlikely to be stem cell–related adverse events and were instead attributed to surgery-related adverse events. Three patients developed mild delirium that lasted between 24 hours and 5 days, which was assumed to be postoperative delirium.

**Table 2:** Adverse events that were observed in at least one subject during the 12-wk follow-up period (all causalities)

Events	Low dose (3.0 × 10^6^ cells), n = 3	High dose (6.0 × 10^6^ cells), n = 6	Total, n = 9
Gastrointestinal disorders, n (%)	0 (0.0)	4 (66.7)	4 (44.4)
Nausea	0 (0.0)	2 (33.3)	2 (22.2)
Colonic polyp	0 (0.0)	1 (16.7)	1 (11.1)
Vomiting	0 (0.0)	1 (16.7)	1 (11.1)
Procedural complications, n (%)	3 (100.0)	6 (100.0)	9 (100.0)
Wound pain	3 (100.0)	6 (100.0)	9 (100.0)
Ligament sprain	0 (0.0)	1 (16.7)	1 (11.1)
Nervous system disorders, n (%)	1 (33.3)	4 (66.7)	5 (55.6)
Headache	1 (33.3)	3 (50.0)	4 (44.4)
Dizziness	0 (0.0)	3 (50.0)	3 (33.3)
Psychiatric disorders, n (%)	0 (0.0)	3 (50.0)	3 (33.3)
Delirium	0 (0.0)	3 (50.0)	3 (33.3)
Others, n (%)	1 (33.3)	1 (16.7)	2 (22.2)
Back pain	1 (33.3)	1 (16.7)	2 (22.2)
Asthenia	1 (33.3)	0 (0.0)	1 (11.1)

Brain MRIs taken 12 weeks and 24 months after the injections confirmed that there were no structural abnormalities including tumor and subdural hemorrhages. Furthermore, no dose-limiting toxicities were evident for either the low- or high-dose hUCB-MSC groups. Results of the mixed lymphocyte reaction performed at 12 weeks and 12 months after injection showed that all nine subjects were immunologically stable.

### 3.2 Clinical and laboratory outcome measures

The changes in ADAS-Cog score from baseline to week 12 and month 24 in the low-dose group were 5.3 ± 3.5 and 20.0 ± 9.9 points, respectively, whereas those in the high-dose group were 3.5 ± 5.6 and 8.6 ± 13.1 points, respectively (Table 3). The changes in S-IADL score from baseline to week 12 and month 24 were 1.7 ± 4.0 and 19.5 ± 6.4 points for the low-dose group and 1.2 ± 5.9 and 12.0 ± 6.0 points for the high-dose group, respectively (Table 3). In addition, the changes in MMSE score from baseline to week 12 and month 24 were −1.7 ± 0.6 and −9.5 ± 0.7 points for the low-dose group and 0.5 ± 2.1 and −8.4 ± 5.6 points for the high-dose group, respectively (Table 3).

**Table 3:** Changes in ADAS-Cog, S-IADL, and MMSE scores before and after the hUCB-MSC injection

	ADAS-Cog			S-IADL			MMSE		
	Low dose	High dose	Total	Low dose	High dose	Total	Low dose	High dose	Total
Screening									
Mean ± SD	38.7 ± 18	28.0 ± 8.6	31.6 ± 12.5	22.0 ± 12.3	22.8 ± 7.9	22.6 ± 8.8	15.0 ± 7.0	17.5 ± 2.9	16.7 ± 4.4
Median	47	29.5	30	17	21	19	12	18	18
Min to max	18 to 51	15 to 37	15 to 51	13 to 36	12 to 32	12 to 36	10 to 23	12 to 20	10 to 23
Week 4									
Mean ± SD	38.7 ± 17.4	30.5 ± 8.3	33.2 ± 11.6	24.0 ± 13.5	23.2 ± 5.8	23.4 ± 8.1	15.7 ± 5.7	16.0 ± 4.0	15.9 ± 4.3
Median	45	29	29	20	24.5	24	14	16.5	16
Min to max	19 to 52	21 to 45	19 to 52	13 to 39	12 to 28	12 to 39	11 to 22	9 to 21	9 to 22
Week 12									
Mean ± SD	44.0 ± 18.2	31.5 ± 10.9	35.7 ± 14	23.7 ± 11.7	24.0 ± 7.9	23.9 ± 8.6	13.3 ± 6.7	18.0 ± 4.1	16.4 ± 5.2
Median	53	32.5	33	19	24.5	24	10	18	18
Min to max	23 to 56	14 to 43	14 to 56	15 to 37	14 to 34	14 to 37	9 to 21	11 to 24	9 to 24
Month 24									
Mean ± SD	54.5 ± 13.4	39.2 ± 10.8	43.6 ± 12.8	34.5 ± 3.5	37.0 ± 6.7	36.3 ± 6.0	8.0 ± 7.1	8.6 ± 7.6	8.4 ± 6.9
Median	54.5	46	46	34.5	41	37	8	12	12
Min to max	45 to 64	23 to 48	23 to 64	32 to 37	29 to 44	29 to 44	3 to 13	0 to 17	0 to 17
Mean change from baseline to week 12									
Mean ± SD	5.3 ± 3.5	3.5 ± 5.6	4.1 ± 4.8	1.7 ± 4.0	1.2 ± 5.9	1.3 ± 5.1	−1.7 ± 0.6	0.5 ± 2.1	−0.2 ± 2.0
Median	5.0	3.5	4.0	1.0	2.0	2.0	−2.0	0.5	−1.0
Min to max	2 to 9	−3 to 13	−3 to 13	−2 to 6	−8 to 8	−8 to 8	−2 to −1	−2 to 4	−2 to 4
Mean change from baseline to month 24									
Mean ± SD	20.0 ± 9.9	8.6 ± 13.1	11.9 ± 12.7	19.5 ± 6.4	12.0 ± 6.0	14.1 ± 6.7	−9.5 ± 0.7	−8.4 ± 5.6	−8.7 ± 4.7
Median	20	12	13	19.5	11	12	−9.5	−6	−9
Min to max	13 to 27	−14 to 18	−14 to 27	15 to 24	6 to 22	6 to 24	−10 to 9	−17 to 3	−17 to 3

Abbreviations: ADAS-Cog, Alzheimer's disease assessment scale-cognitive subscale; S-IADL, Seoul instrumental activities of daily living; MMSE, mini-mental state examination; hUCB-MSC, human umbilical cord blood–derived mesenchymal stem cell; SD, standard deviation.

Changes in the incidence of neuropsychiatric symptoms are shown in Supplementary Table 1. Biomarker changes such as amyloid burden measured by PiB-PET, glucose metabolism measured by FDG-PET, and CSF biomarker level are demonstrated in Supplementary Fig. 1. Although our small sample size precluded statistical analysis, amyloid burden levels in the hUCB-MSC–treated right precuneus did not differ from those of the untreated left precuneus (Supplementary Fig. 1).

## 4 Discussion

This phase 1 clinical trial demonstrated that surgical stereotactic administration of a low (3.0 × 10^6^ cells/60 μL) or high dose (6.0 × 10^6^ cells/60 μL) of hUCB-MSCs into the hippocampus and precuneus is feasible, safe, and well tolerated in patients with mild-to-moderate AD dementia.

Our study is the first, reported so far, to demonstrate the safety of hUCB-MSC injections into the hippocampus and precuneus by stereotactic surgery. We were especially concerned about whether the patients might experience fever, as this has been a frequently reported adverse event in MSC clinical trials [23]. However, none of the nine patients experienced fever. Although some patients experienced wound pain, headache, dizziness, and delirium, there were no reports of serious adverse events related to stem cell treatment. Indeed, a meta-analysis of MSC clinical trials showed that MSC therapy does not increase risk of acute infusional toxicity, arrhythmia, cardiac dysfunction, gastrointestinal or renal dysfunction, infection, death, or tumor development [23]. Thus, the surgical procedure involving the hippocampal and precuneus injections of hUCB-MSCs can be considered safe. The results of this present study may pave a new road for future AD studies.

There were no trends with respect to changes in the AD pathophysiological process as measured by PiB-PET. We expected to see a decrease of PiB SUVR values at the sites injected with hUCB-MSCs, as *in vitro* and *in vivo* animal studies have consistently shown the neuroprotective effects of hUCB-MSCs against amyloid. Indeed, a study showed that hUCB-MSCs protect against Aß42-induced cell death by secreting galectin-3 [3]. Likewise, another study reported that a single injection of hUCB-MSCs into the hippocampus of AD mice decreases the formation of Aß42 plaques in the hippocampus and other regions, which is associated with the migration of hUCB-MSCs toward Aß deposits [2]. However, our single injection of hUCB-MSCs into the human brain did not replicate findings from the animal studies. The discrepancy between animal experiments and our human trial may be explained by several possibilities. First, PiB-PET may not be sufficiently sensitive to detect soluble amyloid or diffuse amyloid plaques [24]. Reduction of amyloid burden after MSC administration in mice was detected through biochemical analysis including immunohistochemical staining, enzyme-linked immunosorbent assay, and Western blot, which can detect soluble and insoluble amyloid as well as different types of amyloid plaques, whereas in our study, changes in amyloid were measured by PiB-PET, which may only detect neuritic amyloid plaques [24]. Second, differences in AD microenvironment between humans and mouse models may explain the different responses to hUCB-MSCs. For example, the APP-PS1 mice used in our previous animal experiments show amyloid accumulation without neuronal loss or inflammation in the brain, whereas humans show both amyloid and tau pathologies with consequent neuronal loss and inflammation. Moreover, prior autopsy studies indicated that more than 50% of clinically diagnosed AD patients harbor other mixed pathologies in addition to amyloid plaques and tau tangles [25], [26]. Therefore, different pathologic environments of the brain may explain the different responses to MSC treatment. As a third possible explanation, mouse AD models rely on xenogeneic transplantation, whereas human trials involved allogeneic transplantation. Thus, with respect to phylogeny, it is possible that lower-order species may benefit more when given MSCs from higher-order species than vice versa. Finally, the absence of an effect may have been due to the fact that the sample size was too small to demonstrate statistical significance.

With respect to treatment efficacy, the lack of controls and small sample size preclude any conclusion. However, the rate of cognitive decline (nine-point drop in MMSE within 2 years) was faster than typical AD (three-point drop in MMSE per year) [27]. We assume that this is because seven of nine participants were early onset AD, which is known to progress faster than typical late-onset AD [28]. Indeed, different rates of progression have been reported among patients with aggressive form of AD showing a decrease in MMSE score six points per year [29], [30].

This study has several limitations. First, because this study had an open-label design with no sham surgery control group and a small number of participants, we could not confirm the efficacy. Second, although we used a stereotaxic injection method, the possibility of hUCB-MSCs being injected into the CSF or subdural space cannot be excluded. However, the error of intraoperative navigation is reported to be up to 2 mm, and we confirmed that the cannula tip was not placed in CSF or blood vessel by verifying no CSF or blood leak through the cannula. Third, passing the needle longitudinally through the hippocampus or precuneus might cause iatrogenic damage. However, to minimize such damage, we used a cannula that had a blunt stylet within the lumen to avoid coring when being passed through the parenchyma.

## 5 Conclusions

We conclude that stereotactic administration of hUCB-MSCs into the hippocampus and precuneus is feasible, safe, and well tolerated. We believe that our study paves the way for additional cell therapy studies. The remaining challenge is to determine if hUCB-MSC injections provide clinical benefit by either slowing or even revising the progression of AD symptoms. Further clinical trials with placebo controls, larger sample size, and a long-term follow-up period are warranted to test the efficacy of this treatment. In addition, new and effective protocols should be developed to augment the MSC effects on AD pathology and to allow repeated injections of hUCB-MSCs because the survival time of hUCB-MSCs injected in the mouse brain is reported to be approximately 4 weeks [2].

**Research in context**

1. Systematic review: The authors reviewed the literature using traditional (e.g., PubMed) sources and meeting abstracts and presentations. Preclinical studies suggest that human umbilical cord blood–derived mesenchymal stem cells (hUCB-MSCs) are beneficial in Alzheimer's disease (AD) mice. Several human clinical trials have suggested that MSCs are effective in slowing down the course of neurodegenerative diseases such as Parkinson's disease, multiple system atrophy, and amyotrophic lateral sclerosis. However, to the best of our knowledge, there has been no clinical trial that has attempted to treat human AD by using MSCs.
2. Interpretation: This phase 1 clinical trial suggests that administration of hUCB-MSCs into the hippocampus and precuneus by stereotactic injection is feasible, safe, and well tolerated.
3. Future directions: Further clinical trials with placebo controls, larger sample size, and a long-term follow-up period are warranted to test the efficacy of this treatment.
