# Supplementary material for: Stereotactic brain injection of human umbilical cord blood mesenchymal stem cells in patients with Alzheimer's disease dementia: A phase 1 clinical trial
Source: Alzheimers Dement (N Y). 2015 Jul 26;1(2):95–102. doi: 10.1016/j.trci.2015.06.007 (PMC5975048; doi:10.1016/j.trci.2015.06.007)
Supplement: Supplementary Data [file mmc1.doc]

**Supplementary Methods**

**PiB-PET acquisition**

All patients underwent [^11^C]PiB-PET scan at Samsung Medical Center using a Discovery STe PET/CT scanner (GE Medical Systems, Milwaukee, WI, USA) in a 3-dimensional scanning mode that examined 35 slices of 4.25-mm thickness spanning the entire brain. [^11^C]PiB was injected into an antecubital vein as a bolus injection with a mean dose of 420 MBq (i.e., range 259–550 MBq). 60 minutes after injection, a CT scan was performed for attenuation correction. A 30-minute emission static PET scan was then initiated. Attenuation corrected PET images were reconstructed from the CT data using an iterative reconstruction method*.* The specific radioactivity of [^11^C]PiB at the time of administration was higher than 33.3 GBq/μmol for patients. In all PET studies, the radiochemical purity of the radiotracer was higher than 95%.

**PiB-PET data analysis**

We co-registered PiB-PET images to individual MRIs, which were normalized to a T1-weighted MRI template. Using these parameters, MRI co-registered PiB-PET images were normalized to the MRI template. We obtained the quantitative regional values of PiB retention on the spatially normalized PiB images, by an automated VOI analysis using the automated anatomical labeling (AAL) atlas. We processed data using SPM Version 5 (SPM5) within Matlab 6.5 (MathWorks, Natick, MA, USA).

To measure PiB retention, we used the cerebral cortical region to determine the cerebellum uptake ratio. We used the cerebellar gray matter as a reference region. We selected 28 cortical VOIs from the left and right hemispheres using the AAL atlas. The cerebral cortical VOIs that were chosen for this study consisted of the bilateral frontal (superior and middle frontal gyri, the medial portion of superior frontal gyrus, the opercular portion of the inferior frontal gyrus, the triangular portion of the inferior frontal gyrus, supplementary motor area, orbital portion of the superior, middle, and inferior orbital frontal gyri, and rectus and olfactory cortex), posterior cingulate gyri, parietal (superior and inferior parietal, supramarginal and angular gyri, and precuneus), lateral temporal (superior, middle and inferior temporal gyri, and heschl gyri), and occipital (superior, middle, and inferior occipital gyri, cuneus, calcarine fissure, and lingual and fusiform gyri). Regional cerebral cortical uptake ratios were calculated by dividing each cortical VOI uptake ratio by the mean uptake of the cerebellar cortex (cerebellum crus1 and crus2). The PiB standardized uptake value ratio (SUVR) was calculated from the volume-weighted average uptake ratio of 28 bilateral cerebral cortical VOIs. We defined the PiB SUVR as a continuous variable representing the amyloid burden.

**FDG-PET**

We measured the resting state regional brain glucose metabolism using 2-[^18^F]fluoro-2-deoxy-D-glucose ([18F]FDG). The participants fasted at least 6 hours before the scan. After intravenous injection of 4.8 MBq/kg FDG using a GE Advance PET scanner, thirty-minute PET scans were acquired 40 minutes. In order to minimize external stimuli during the FDG uptake period, the participants were situated in a dimly lit room with their eyes closed. At 30 minutes after intravenous injection of 4.8 MBq/kg [18F]FDG, CT scan of the entire brain was performed with a continuous spiral technique with an 8-slice helical CT. After the CT scan, an emission PET scan was obtained for 7 minutes in 3-dimensional mode using a GE Advance PET scanner. Attenuation-corrected PET images (voxel size, 1.95 x 1.95 x 4.25 mm) were reconstructed from the CT data using an ordered-subset expectation maximization algorithm.

**SPM Analysis of Regional Glucose Metabolism**

We analyzed PET images using SPM2 (WellcomeDepartment of Cognitive Neurology, Institute of Neurology, London, UK) [1] and we implemented PET images using Matlab 7.0 (MathWorks Inc.). In order to remove inter-subject anatomical variability, all of the images were spatially normalized into the MNI standard template (Montreal Neurological Institute, McGill University, Montreal, Canada). Spatially normalized images were smoothed with a 12 mm FWHM isotropic Gaussian kernel. The count of each voxel was normalized to the average count of the cerebellum with proportional scaling in SPM5.

**eTable 1. Number of patients experiencing neuropsychiatric symptoms**

|  | Delusion | Hallucination | Agitation | Depression | Anxiety | Euphoria | Apathy | Disinhibition | Irritability | Abberant motor | Sleep | Appetite |
| --- | --- | --- | --- | --- | --- | --- | --- | --- | --- | --- | --- | --- |
| Screening | 2 (0, 2) | 1 (0, 1) | 5 (3, 2) | 5 (2, 3) | 6 (2, 4) | 2 (0, 2) | 7 (3, 4) | 4 (0, 4) | 8 (2, 6) | 4 (1, 3) | 2 (0, 2) | 3 (1, 2) |
| Week 4 | 3 (0, 3) | 1 (0, 1) | 2 (0, 2) | 3 (1, 2) | 5 (3, 2) | 0 (0, 0) | 5 (2, 3) | 1 (0, 1) | 4 (2, 2) | 1 (0, 1) | 1 (0, 1) | 3 (2, 1) |
| Week 12 | 2 (0, 2) | 1 (0, 1) | 5 (1, 4) | 6 (3, 3) | 4 (2, 2) | 1 (0, 1) | 5 (2, 3) | 1 (0, 1) | 4 (2, 2) | 2 (0, 2) | 4 (0, 4) | 1 (1, 0) |
| Month 24 | 4 (1, 3) | 2 (1, 1) | 5 (2, 3) | 3 (1, 2) | 3 (1, 2) | 0 (0, 0) | 6 (2, 4) | 3 (2, 1) | 3 (2, 1) | 7 (2. 5) | 3 (1, 2) | 5 (2, 3) |

Numbers shown, total (low dose, high dose)

eTable 1. Changes in ADAS-cog, S-IADL, and MMSE score before and after the hUCB-MSC injection

|  |  |  | | |  |  | | |  |  | | |
| --- | --- | --- | --- | --- | --- | --- | --- | --- | --- | --- | --- | --- |
|  | |  |  |  |  |  |  |  |  |  |  |  |
|  | | | |  |  |  |  |  |  |  |  |  |
|  |  |  |  |  |  |  |  |  |  |  |  |  |
|  |  |  |  |  |  |  |  |  |  |  |  |  |
|  |  |  |  |  |  |  |  |  |  |  |  |  |
|  | | | |  |  |  |  |  |  |  |  |  |
|  |  |  |  |  |  |  |  |  |  |  |  |  |
|  |  |  |  |  |  |  |  |  |  |  |  |  |
|  |  |  |  |  |  |  |  |  |  |  |  |  |
|  | | | |  |  |  |  |  |  |  |  |  |
|  |  |  |  |  |  |  |  |  |  |  |  |  |
|  |  |  |  |  |  |  |  |  |  |  |  |  |
|  |  |  |  |  |  |  |  |  |  |  |  |  |
|  | | | |  |  |  |  |  |  |  |  |  |
|  |  |  |  |  |  |  |  |  |  |  |  |  |
|  |  |  |  |  |  |  |  |  |  |  |  |  |
|  |  |  |  |  |  |  |  |  |  |  |  |  |


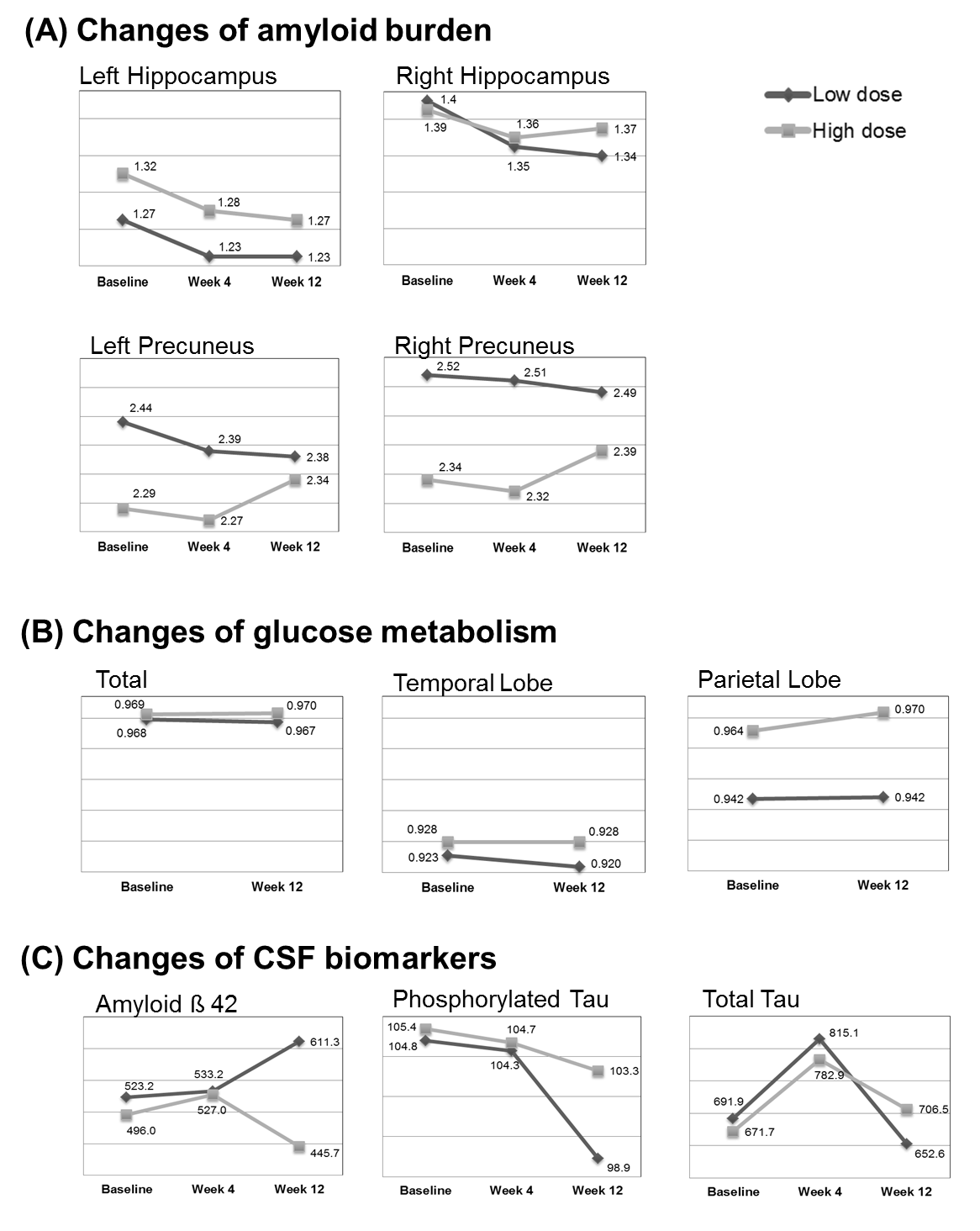


**eFigure 1. Changes in amyloid burden measured by PiB SUVR (A), glucose metabolism measured by CMRglc (B), and cerebrospinal fluid (CSF) biomarker level measurement (C) over a 12 week follow-up after human umbilical cord blood derived mesenchymal stem cells (hUCB-MSCs) injection.** PiB SUVR, Pittsburg Compound B standardized uptake value ratio; CMRglc, cerebral metabolic rate for glucose consumption

**Reference**

[1] K.J. Friston, J. Ashburner, C.D. Frith, J.B. Poline, J.D. Heather, R.S.J. Frackowiak, Spatial registration and normalization of images. Human Brain Mapping 1995;3:165-189.
